# Clinical Assessment of Lamina Cribrosa Curvature in Eyes with Primary Open-Angle Glaucoma

**DOI:** 10.1371/journal.pone.0150260

**Published:** 2016-03-10

**Authors:** Yong Woo Kim, Jin Wook Jeoung, Dai Woo Kim, Michael J. A. Girard, Jean Martial Mari, Ki Ho Park, Dong Myung Kim

**Affiliations:** 1 Department of Ophthalmology, Armed Forces Busan Hospital, Busan, Korea; 2 Department of Ophthalmology, Seoul National University Hospital, Seoul National University College of Medicine, Seoul, Korea; 3 Department of Ophthalmology, Kim’s Eye Hospital, Seoul, Korea; 4 Department of Biomedical Engineering, National University of Singapore, Singapore, Singapore; 5 Singapore Eye Research Institute, Singapore, Singapore; 6 University of French Polynesia, Tahiti, French Polynesia; Medical University Graz, AUSTRIA

## Abstract

**Purpose:**

Quantitative evaluation of lamina cribrosa (LC) posterior bowing in primary open-angle glaucoma (POAG) eyes using swept-source optical coherence tomography.

**Methods:**

Patients with POAG (*n* = 123 eyes) and healthy individuals of a similar age (*n* = 92 eyes) were prospectively recruited. Anterior laminar insertion depth (ALID) was defined as the vertical distance between the anterior laminar insertion and a reference plane connecting the Bruch’s membrane openings (BMO). The mean LC depth (mLCD) was approximated by dividing the area enclosed by the anterior LC, the BMO reference plane, and the two vertical lines for ALID measurement by the length between those two vertical lines. The LC curvature index was defined as the difference between the mLCD and the ALID. The factors influencing the LC curvature index were evaluated.

**Results:**

The ALID and mLCD were significantly larger in POAG eyes than in healthy controls (*P* < 0.05). The LC curvature index was significantly larger in POAG eyes than in healthy controls on both the horizontal (85.8 ± 34.1 vs. 68.2 ± 32.3 μm) and vertical meridians (49.8 ± 38.5 vs. 32.2 ± 31.1 μm, all *P <* 0.001). Multivariate regression showed significant associations of greater disc area (*P* < 0.001), vertical C/D ratio (*P* < 0.001) and mLCD (*P* < 0.001), smaller rim area (*P* = 0.001), thinner average RNFLT (*P* < 0.001), and myopic refraction (*P* = 0.049) with increased LC curvature index. There was no difference in the LC curvature index between mild (MD > –6 dB) and moderate-to-advanced glaucoma (MD < –6 dB, *P* = 0.95).

**Conclusions:**

LC posterior bowing was increased in POAG eyes, and was significantly associated with structural optic nerve head (ONH) changes but not with functional glaucoma severity. Quantitative evaluation of LC curvature can facilitate assessment of glaucomatous ONH change.

## Introduction

The lamina cribrosa (LC), where retinal nerve fiber bundles exit from the eye, plays a prominent role in the pathogenesis of glaucoma according to the biomechanical theory.[1–3] The LC deformations caused by increased intraocular pressure (IOP), in particular, deformations involving posterior bowing of the LC and posterior displacement of the laminar insertion, have been widely studied in experimental animal eyes as well as ex-vivo human eyes.[4–8] With the advent of enhanced depth imaging (EDI) spectral domain optical coherence tomography (SD-OCT) and swept-source OCT (SS-OCT), in vivo evaluation of the LC deformation in glaucoma eyes has generated considerable interest.[9–14]

The LC depth and the LC thickness have been used as representative surrogate markers for LC deformation in previous in vivo LC studies using OCT. These parameters have enabled quantitative and objective evaluations of LC structure in both a cross-sectional and a longitudinal manner. Glaucomatous optic nerve heads typically present with a deeper[15] but thinner LC[16] compared to healthy controls. It is well documented that the LC depth decreases and the LC thickness increases with IOP-lowering treatments.[17–20] However, LC depth provides information only on the ‘position of LC’ referenced to a certain anatomical landmark (usually the Bruch’s membrane opening [BMO] plane). In addition, the “LC thickness” measurements from OCT should be interpreted with caution since the posterior border of the LC is not always clearly visible and it has never been validated by concurrent histological analysis.[9, 12, 21]

Posterior bowing of the LC may be related to mechanical or vascular damage to the optic nerve head (ONH), including the ganglion cell axons.[1] It may be reasonably hypothesized that the greater the posterior bowing of the LC, the greater the burden to retinal ganglion cell axons. Development of a novel parameter characterizing ‘anterior LC curvature’ (additionally to LC ‘depth’ and ‘thickness’) deserves clinical interest. We propose a novel and simple index, termed the “LC curvature index,” for quantitative evaluation of LC posterior bowing. The LC curvature index is calculated as the difference between the mean LC depth (mLCD) and anterior laminar insertion depth (ALID). The purpose of the present study is to quantitatively evaluate LC posterior bowing (LC curvature index) in both POAG and healthy eyes, and to investigate the factors associated with increased LC posterior bowing (LC curvature index).

## Methods

The subjects of the present study comprised glaucoma patients and healthy individuals with similar age from the *S*wept-Source *O*ptical Coherence Tomography *S*tudy of *L*amina *C*ribrosa (*SOS-LC*), an ongoing prospective study at Seoul National University Hospital (SNUH). They were consecutive subjects who met the eligibility criteria and provided written informed consent for participation. The present study was approved by the Seoul National University Hospital Institutional Review Board and followed the tenets of the Declaration of Helsinki (1964).

### Study subjects

The subjects who were enrolled in the *SOS-LC* underwent a complete ophthalmic examination, including a visual acuity assessment, slit-lamp biomicroscopy, gonioscopy, Goldmann applanation tonometry, refractions, dilated fundus examination, disc stereophotography, and red-free fundus photography by a digital fundus camera (VX-10, Tokyo, Japan), as well as standard automated perimetry (Humphrey C 30–2 SITA-Standard visual field; Carl Zeiss Meditec, Inc., Dublin, CA). The central corneal thickness (Pocket II; Quantel Medical, Clermont-Ferrand, France), axial length (AXIS-II Ultrasonic Biometer; Quantel Medical S.A., Bozeman, MT), disc area and average peripapillary retinal nerve fiber layer (RNFL) thickness (Cirrus HD-OCT, Carl Zeiss Meditec, Dublin, CA) were measured.

To be included in the *SOS-LC*, glaucoma patients were required to satisfy the definition of POAG, including the presence of glaucomatous optic disc changes such as focal notching and thinning, RNFL defects on disc stereophotography and red-free fundus photography, a glaucomatous VF defect, and an open angle confirmed by gonioscopic examination. A glaucomatous VF defect was defined as (1) glaucoma hemifield test values outside the normal limits or (2) three or more abnormal points with a probability of *P* < 0.05, of which at least one point has a pattern deviation of *P* < 0.01, or (3) a pattern standard deviation of *P* < 0.05. The visual field defects were confirmed on two consecutive reliable tests (fixation loss rate ≤ 20%, false-positive and false-negative error rates ≤ 25%).

The healthy individuals with similar age were those who had visited the SNUH outpatient clinic for regular ocular check-ups (e.g., dry eye, cataract) and showed no abnormalities on disc stereophotography, red-free fundus photography, and standard automated perimetry. The untreated IOP value was defined as the mean of two measurements before IOP-lowering management was initiated.

The present study excluded subjects with (1) a history of intraocular surgery including glaucoma surgery, (2) a history of intraocular disease (e.g., proliferative diabetic retinopathy, retinal vein occlusion), or (3) OCT scans with poor visualization of the peripheral LC due to vascular shadowing or peripheral focal LC defects.

### Swept-Source Optical Coherence Tomography Imaging of the Optic Disc

All of the participants had been scanned with the DRI OCT-1 Atlantis 3D SS-OCT device (Topcon Medical Systems, Oakland, NJ). Five-line cross-scans (five lines horizontal and five lines vertical) centered at the optic disc with 0.25-mm spacing between the cross-lines and a 6.0-mm scan width were performed. A total of 32 A-scans were averaged for each line of five cross-lines. The central three out of five cross-line scans (total 579 horizontal and 579 vertical scans of 193 subjects) were selected, and the mean measurements of the three scans were used for the analysis. Of these, 42 scans (20 horizontal and 22 vertical scans) were excluded because of poor OCT scan quality that did not allow clear visualization of the peripheral LC (i.e., severe vascular shadowing). Therefore, a total of 559 horizontal scans and 557 vertical scans were included to the analysis.

To enhance the visibility of the peripheral LC, adaptive compensation was applied to all scan images according to the previously published protocols.[21–23]

### Measurement of ALID, mLCD, and LC curvature index

**Fig 1:**
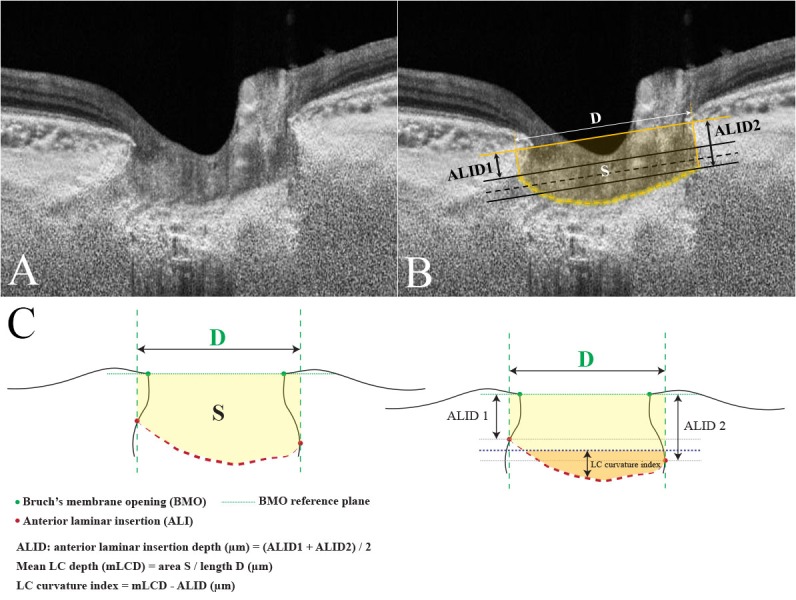
Measurement of the lamina cribrosa (LC) curvature index using swept-source optical coherence tomography (SS-OCT) scans. The optic disc scan performed by SS-OCT without (A) and with (B) guidelines. The line connecting the two Bruch’s membrane openings (BMOs) is selected as the reference plane. The anterior LC surface has been manually depicted as a yellow dotted line (B). The two vertical lines are drawn from the anterior laminar insertion (ALI) to the BMO reference plane. The distance between the two cross-points is measured as *D*. The two black solid lines are drawn parallel to the BMO reference plane at each ALI, and the black dotted line indicates the mean level of the ALI. This corresponds to the mean anterior laminar insertion depth (ALID). The area surrounded by the anterior LC surface, BMO reference plane, and the two vertical lines is measured as *S*. The mean LC depth (mLCD) is computed by dividing *S* by *D*. The LC curvature index is the difference between the mLCD and mean ALID. The schematic diagram is demonstrated in Fig 1C.

All of the measurements were performed by using ImageJ software (developed by Wayne Rasband, National Institutes of Health, Bethesda, MD; available at http://imagej.nih.gov/ij/). The ALID was defined as the vertical distance between the anterior laminar insertion (ALI) and the reference plane connecting the BMO. The mean values of temporal and nasal ALID and that of the superior and inferior ALID were defined as the horizontal and vertical ALID, respectively. The area enclosed by the anterior laminar surface, the two vertical lines for the ALID measurement, and the BMO reference plane was measured. The mLCD was computed by dividing this area by the length between the two cross-points made by vertical lines drawn from the ALI to the BMO reference plane. The anterior LC surface was manually depicted as if there was no discontinuity on the anterior LC border, including vascular shadowing or LC pores. The horizontal and vertical mLCD were measured on each horizontal and vertical scan. The horizontal and vertical LC curvature index measures were defined as the difference between mLCD and ALID (mLCD–ALID) on each horizontal and vertical scan, respectively. Thus, a higher LC curvature index indicated increased posterior bowing of the LC (**Fig 1**). The mean of the horizontal and vertical LC curvature index measurements was defined as the overall LC curvature index. An experienced ophthalmologist (Y.W.K.) who was masked to the subjects’ clinical information performed the measurements.

### Statistical analysis

To evaluate the intraobserver and interobserver reproducibility of the horizontal and vertical LC vault respectively, two observers (Y.W.K. & D.W.K.) blind to the clinical information performed measurements in 20 randomly selected B-scans. The analysis was based on three independent series of re-evaluations; the absolute agreement of a single observer’s measurements and the mean of all three measurements of the two observers was calculated with the intraclass correlation coefficient (ICC) obtained from a 2-way mixed-effect model. The continuous variables were compared using Student’s *t*-test or one-way analysis of variance (ANOVA) with Scheffé’s post-hoc test, and the categorical variables were compared using a chi-square test. The general linear model was used to determine the factors (age, gender, diabetes mellitus, hypertension, untreated IOP, IOP at examination, central corneal thickness [CCT], spherical equivalence [SE], axial length [AXL], disc area, rim area, vertical C/D ratio, average RNFL thickness, mean deviation [MD] of visual field [VF], and mLCD) associated with increased overall LC curvature index, first with an univariate model, and then with a multivariate model that included the univariate model variables with *P* < 0.10. Statistical analyses were performed with the Statistical Package for Social Sciences version 21.0 for Windows (SPSS, Inc., Chicago, IL). The data obtained are presented as mean ± standard deviation values, and the level of statistical significance was set at *P* < 0.05.

## Results

### Baseline characteristics

The present study included 123 eyes of 123 POAG patients (20 preperimetric and 103 perimetric glaucoma patients) and 92 eyes of 92 healthy individuals with similar age. There were no differences in underlying disease (i.e., diabetes and hypertension), refraction, AXL, and disc area between the two groups (**Table 1**). The healthy control included more females (*P* = 0.040). The untreated IOP was significantly larger in POAG eyes compared to healthy control (15.5 ± 3.7 mmHg vs. 13.7 ± 2.9 mmHg, *P* < 0.001). Since the POAG patients were then placed under intensive IOP-lowering treatment, their IOP at examination was lower than the healthy controls’ (12.3 ± 2.3 mmHg vs. 13.2 ± 2.8 mmHg, *P* = 0.008). In addition, the rim area (*P* < 0.001), vertical C/D ratio (*P* < 0.001), average RNFL thickness (*P* < 0.001), MD of VF (*P* < 0.001), and CCT (*P* = 0.027) were all significantly lower in POAG eyes compared to healthy controls (**Table 1**).

**Table 1:** Subject demographics.

Variable	POAG (*n* = 123 eyes)	Healthy (*n* = 92 eyes)	P-value
Age, year	60.4 ± 10.6	62.1 ± 10.7	0.23*
**Female, *n* (%)**	**56 (45.5)**	**55 (59.8)**	**0.040**†
Diabetic mellitus, *n* (%)	12 (10.7)	14 (15.2)	0.40†
Hypertension, *n* (%)	31 (27.7)	30 (32.6)	0.45†
**Untreated IOP, mmHg**	**15.5 ± 3.7**	**13.7 ± 2.9**	<0.001*
**IOP at examination, mmHg**	**12.3 ± 2.3**	**13.2 ± 2.8**	0.011*
SE, D	–1.08 ± 2.51	–0.61 ± 2.67	0.21*
AXL, mm	24.13 ± 1.16	23.80 ± 1.28	0.11*
**Central corneal thickness, μm**	**529.3 ± 33.8**	**540.4 ± 30.7**	0.027*
Disc area, mm^2^	2.03 ± 0.42	2.14 ± 0.36	0.07*
**Rim area, mm**^**2**^	**0.86 ± 0.22**	**1.14 ± 0.24**	<0.001*
**Vertical C/D ratio**	**0.74 ± 0.11**	**0.63 ± 0.11**	<0.001*
**Average RNFL thickness, μm**	**73.8 ± 12.0**	**89.4 ± 9.7**	<0.001*
**MD, dB**	**–4.59 ± 4.32**	**–0.65 ± 1.98**	<0.001*

POAG: primary open-angle glaucoma, IOP: intraocular pressure, SE: spherical equivalence, D: diopter, AXL: axial length, MD: mean deviation, RNFL: retinal nerve fiber layer

Mean ± standard deviation

*Comparison performed using Student’s t-test

†Comparison performed using chi-square test

Statistically significant values are shown in bold.

### ALID, mLCD, and LC curvature index

Horizontal LC curvature index measurement by the two observers showed excellent intraobserver (ICC = 0.935 and 95% confidence interval [CI] = 0.738–0.984 for observer 1; ICC = 0.934 and 95% CI = 0.668–0.993 for observer 2) and interobserver (ICC = 0.932 and 95% CI = 0.858–0.968) reproducibility (all *P* < 0.001). Vertical LC curvature index measurement by the two observers also showed excellent intraobserver (ICC = 0.959 and 95% CI = 0.835–0.990 for observer 1; ICC = 0.953 and 95% CI = 0.761–0.995 for observer 2) and interobserver (ICC = 0.927 and 95% CI = 0.845–0.966) reproducibility (all *P* < 0.001).

The horizontal ALID was significantly larger in POAG eyes (374.2 ± 101.2 μm) than in healthy eyes (332.9 ± 97.4 μm, *P* = 0.003). The vertical ALID was also significantly larger in POAG eyes (426.2 ± 102.9 μm) compared to healthy eyes (371.6 ± 109.1 μm, *P* < 0.001, **Table 2**). The horizontal and vertical mLCD were significantly larger in POAG eyes (460.0 ± 109.2 μm and 476.0 ± 104.2 μm, respectively) compared to healthy eyes (401.1 ± 112.1 μm and 403.8 ± 118.7 μm, respectively, all *P* < 0.001, **Table 2**).

**Fig 2:**
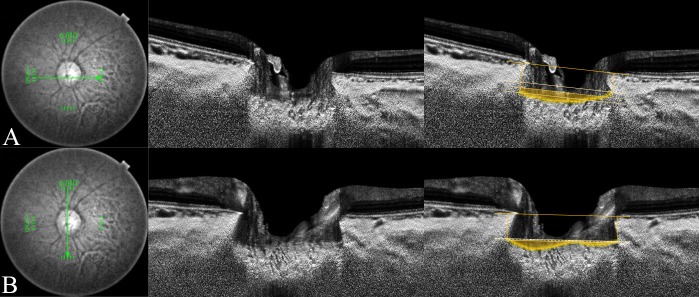
Measurement of the lamina cribrosa (LC) curvature index in healthy eye. Horizontal and vertical optic disc scans of a 53-year-old healthy woman. The image delineated with yellow guidelines is the same as that depicted on the left side. The horizontal and vertical mean anterior laminar insertion depth (ALID) was 238.8 μm and 311.9 μm, respectively. The horizontal and vertical mean LC depth was 278.5 μm and 325.7 μm, respectively. The horizontal LC curvature index was 39.8 μm and the vertical LC curvature index was 13.8 μm. The yellow-shaded area exhibits the degree of the posteriorly located anterior LC surface according to the mean level of the anterior laminar insertion (ALI).

**Fig 3:**
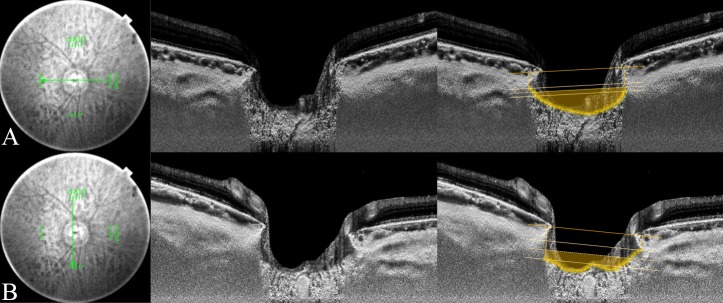
Measurement of lamina cribrosa (LC) curvature index in primary open-angle glaucoma eye. The horizontal and vertical optic disc scans of a 70-year-old patient with primary open-angle glaucoma (POAG). The image delineated with yellow guidelines is the same as that depicted on the left side. The horizontal and vertical mean anterior laminar insertion depth (ALID) was 304.6 μm and 274.1 μm, respectively. The horizontal and vertical mean LC depth was 404.9 μm and 366.4 μm, respectively. The horizontal LC curvature index was 100.3 μm and the vertical LC curvature index was 92.3 μm. The yellow-shaded area exhibits the degree of the posteriorly located anterior LC surface according to the mean level of the anterior laminar insertion (ALI).

The horizontal LC curvature index (85.8 ± 34.1 μm) and the vertical LC curvature index (49.8 ± 38.5 μm) were both significantly larger in POAG eyes than in healthy eyes (68.2 ± 32.3 μm and 32.2 ± 31.1 μm, respectively, all *P* < 0.001, **Table 2**). Representative cases showing the differences in the ALID, mLCD, and LC curvature index between POAG and healthy eyes are featured in **Figs 2 and 3**.

**Table 2:** Comparison of anterior laminar insertion depth (ALID), mean lamina cribrosa depth (mLCD), and lamina cribrosa (LC) curvature index between the eyes with primary open-angle glaucoma and healthy control.

		POAG (*n* = 123 eyes)	Healthy (*n* = 92 eyes)	P-value*
Horizontal	ALID, μm	**374.2 ± 101.2**	**332.9 ± 97.4**	**0.003**
	mLCD, μm	**460.0 ± 109.2**	**401.1 ± 112.1**	**<0.001**
	LC curvature index, μm	**85.8 ± 34.1**	**68.2 ± 32.3**	**<0.001**
Vertical	ALID, μm	**426.2 ± 102.9**	**371.6 ± 109.1**	**<0.001**
	mLCD, μm	**476.0 ± 104.2**	**403.8 ± 118.7**	**<0.001**
	LC curvature index, μm	**49.8 ± 38.5**	**32.2 ± 31.1**	**<0.001**

ALID: anterior laminar insertion depth, LC: lamina cribrosa, mLCD: mean anterior lamina cribrosa surface depth, POAG: primary open-angle glaucoma

Mean ± standard deviation

*Comparison performed using Student’s t-test

Statistically significant values are shown in bold.

### Factors associated with increased overall LC curvature index

In the univariate analysis, the factors associated with increased overall LC curvature index were greater disc area (*P* = 0.002), smaller rim area (*P <* 0.001), greater vertical C/D ratio (*P* < 0.001), decreased average RNFL thickness (*P* < 0.001), and increased mLCD (*P* < 0.001) (**Table 3**). The variables that showed significance at *P* < 0.10, i.e., male gender, untreated IOP, SE, disc area, rim area, vertical C/D ratio, average RNFL thickness, MD of VF, and mLCD were included in the multivariate model. To avoid complications from an interaction between disc area, rim area, vertical C/D ratio, average RNFL thickness and MD of VF, linear regression analysis was performed separately for these variables (**Table 4**). In the multivariate analysis, the SE (model 1: β = –1.3, *P* = 0.049), disc area (model 1: β = 21.3, *P* < 0.001; model 2: β = 23.9, *P* < 0.001), rim area (model 3: β = –27.2, *P* = 0.001), vertical C/D ratio (model 4: β = 63.0, *P* < 0.001; model 5: β = 88.8, *P* < 0.001), average RNFL thickness (model 1: β = –0.7, *P* < 0.001), and mLCD (model 1 to 5:β = 0.1, *P* < 0.001) were all significantly associated with increased overall LC curvature index values (**Table 4**).

**Table 3:** Factors associated with increased lamina cribrosa curvature index (univariate analysis).

Variable	Univariate analysis		
	β	95% CI	P-value
Age, for each year older	–0.2	–0.5, 0.2	0.41
Gender, male	7.3	0.3, 15.0	0.06
DM	6.1	–5.6, 17.8	0.30
HTN	–0.3	–9.0, 8.3	0.94
Untreated IOP, per 1 mmHg increase	1.1	–0.01, 2.2	0.05
IOP at examination, per 1 mmHg increase	0.3	–1.2, 1.8	0.69
SE, per 1 increase	–1.6	–3.2, 0.03	0.06
AXL, per 1 mm increase	2.0	–1.5, 5.6	0.26
CCT, per 1 μm increase	–0.02	–0.1, 0.1	0.81
**Disc area, per 1 mm**^**2**^ **increase**	**14.6**	**5.4, 23.8**	**0.002**
**Rim area, per 1 mm**^**2**^ **increase**	**–51.5**	**–64.7, –38.3**	**<0.001**
**Vertical C/D ratio, per 0.1 increase**	**110.4**	**81.4, 139.4**	**<0.001**
**Average RNFL thickness, per 1 ㎛ increase**	**–0.6**	**–0.9, –0.4**	**<0.001**
MD of VF, per 1 dB increase	–1.0	–2.1, 0.04	0.06
**mLCD, per 1 ㎛ increase**	**0.1**	**0.1, 0.2**	**<0.001**

AXL: axial length, CCT: central corneal thickness, CI: confidence interval, IOP: intraocular pressure, RNFL: retinal nerve fiber layer, MD: mean deviation, VF: visual field

Statistical analysis was performed using the general linear model.

Statistically significant values are shown in bold.

**Table 4:** Factors associated with increased lamina cribrosa curvature index (multivariate analysis).

Variable	Multivariate analysis model 1*			Multivariate analysis model 2*			Multivariate analysis model 3*			Multivariate analysis model 4*			Multivariate analysis model 5*		
	β	95% CI	P-value	β	95% CI	P-value	β	95% CI	P-value	β	95% CI	P-value	β	95% CI	P-value
Gender, male	2.0	–4.1, 8.1	0.52	2.6	–5.1, 10.4	0.50	3.5	–3.0, 10.0	0.29	3.48	–10.0, –3.0	0.29	2.3	–5.7, 10.3	0.57
Untreated IOP, per 1 mmHg increase	–0.03	–0.9, 0.9	0.95	0.6	–0.5, 1.7	0.28	0.04	–1.0, 1.0	0.94	–0.1	–1.0, 0.9	0.88	0.4	–0.7, 1.6	0.45
**SE, per 1 increase**	**–1.3**	**–2.6, –0.004**	**0.049**	–0.7	–2.3, 0.8	0.35	0.03	–1.4, 1.5	0.97	–0.4	–1.7, 1.0	0.58	0.1	–1.5, 1.7	0.90
**Disc area, per 1 mm**^**2**^ **increase**	**21.3**	**14.3, 28.2**	**<0.001**	**23.9**	**15.6, 32.1**	**<0.001**									
**Rim area, per 1 mm**^**2**^ **increase**							**–27.2**	**–43.3, –11.2**	**0.001**						
**Vertical C/D ratio, per 0.1 increase**										**63.0**	**29.3, 96.6**	**<0.001**	**88.8**	**52.4, 125.2**	**<0.001**
**Average RNFL thickness, per 1 ㎛ increase**	**–0.7**	**–0.9, –0.4**	**<0.001**							–0.2	–0.6, 0.1	0.21			
MD of VF, per 1 dB increase				–0.3	–1.3, 0.8	0.62							0.7	–0.4, 1.9	0.21
**mLCD, per 1 ㎛ increase**	**0.1**	**0.1, 0.2**	**<0.001**	**0.1**	**0.1, 0.2**	**<0.001**	**0.1**	**0.06, 0.13**	**<0.001**	**0.1**	**0.08, 0.14**	**<0.001**	**0.1**	**0.07, 0.15**	**<0.001**

AXL: axial length, CCT: central corneal thickness, CI: confidence interval, IOP: intraocular pressure, RNFL: retinal nerve fiber layer, MD: mean deviation, VF: visual field

Statistical analysis was performed using the general linear model. Statistically significant values are shown in bold.

* Factors with P < 0.10 in the univariate analysis were included in the multivariate analysis.

### Difference of LC curvature index according to glaucoma severity

**Fig 4:**
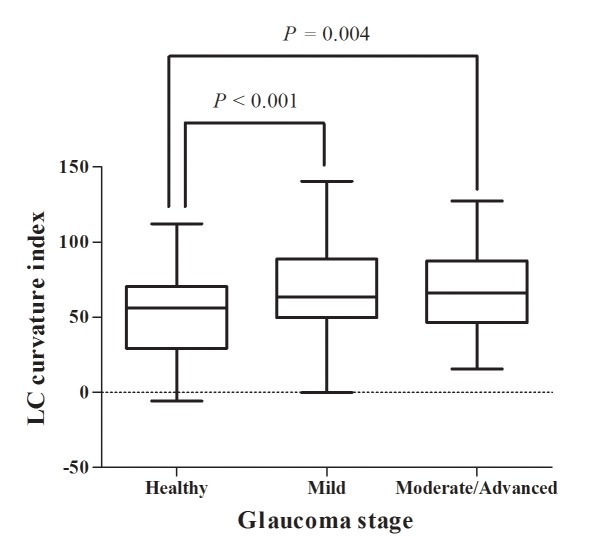
Differences of LC curvature index according to glaucoma stage. Eyes with mild (n = 92, MD > -6 dB) and moderate-to-advanced glaucoma (n = 31, MD < –6 dB) showed an increased LC curvature index relative to the healthy eyes (n = 92, 50.2 ± 26.6 μm, all *P* < 0.05). However, there was no significant difference between mild and moderate-to-advanced glaucoma eyes (67.3 ± 27.3 vs. 69.2 ± 29.0 μm, *P* = 0.95).

The POAG patients were sub-grouped as mild glaucoma (MD > –6 dB, *n* = 92) and moderate-to-advanced glaucoma (MD < –6 dB, *n* = 31). There were no significant differences in age, gender, underlying diseases, AXL or CCT among the healthy control, mild glaucoma and moderate-to-advanced glaucoma groups (**Table 5**). Although the LC curvature index was greater in POAG eyes than in healthy eyes, there was no significant difference between eyes with mild glaucoma (67.3 ± 27.3 μm) and those with moderate-to-advanced glaucoma (69.2 ± 29.0 μm) (*P* = 0.95, **Fig 4**).

**Table 5:** Patient demographics according to the severity of glaucoma.

Variable	Healthy (*n* = 92)	Mild glaucoma (*n* = 92)	Moderate to advanced glaucoma (*n* = 31)	P-value	Post hoc anlaysis
Age, year	62.1 ± 10.7	60.8 ± 10.5	58.9 ± 11.1	0.33*	
Female, *n* (%)	55 (59.8)	41 (44.6)	15 (48.4)	0.11†	
DM, *n* (%)	14 (15.2)	9 (10.6)	3 (11.1)	0.63†	
HTN, *n* (%)	30 (32.6)	25 (29.4)	6 (22.2)	0.58†	
**Untreated IOP, mmHg**	**13.7 ± 2.9**	**15.3 ± 3.8**	**15.9 ± 3.7**	0.001*	**A<B,C**
**IOP at examination, mmHg**	**13.2 ± 2.8**	**12.2 ± 2.2**	**12.5 ± 2.5**	0.028*	**A>B**
AXL, mm	23.8 ± 1.3	24.2 ± 1.2	24.1 ± 1.2	0.27*	
**CCT, μm**	**540.4 ± 30.7**	**531.2 ± 32.2**	**523.4 ± 38.2**	0.049*	**NS**
**Average RNFL thickness, μm**	**89.4 ± 9.7**	**76.1 ± 11.8**	**67.0 ± 9.7**	<0.001*	**A>B>C**
**MD, dB**	**–0.6 ± 2.0**	**–2.5 ± 2.2**	**–10.1 ± 3.5**	<0.001*	**A>B>C**

IOP: intraocular pressure, AXL: axial length, CCT: central corneal thickness, RNFL: retinal nerve fiber layer, MD: mean deviation.

Mean ± standard deviation

* Comparison was performed using one-way analysis of variance with post hoc Scheffe’s multiple comparison testing

†Comparison was performed using chi-square test. Statistically significant values are shown in bold.

## Discussion

The main purpose of the present study was to quantitatively evaluate the degree of LC posterior bowing in POAG and healthy eyes by using SS-OCT. The present study obtained numerical information on anterior LC curvature and demonstrated that the POAG eyes showed increased LC posterior bowing (LC curvature index) in both the horizontal and vertical meridians compared to healthy controls.

On the assumption that the posterior bowing of LC is an indicator of the IOP-related strain at a given level of IOP, we proposed a novel index (LC curvature index) to evaluate LC bowing quantitatively. The LC curvature index may provide additional structural information of LC compared to “LC depth,” a measure that has been used frequently in previous OCT studies. First, the anterior LC is not always an even, bow-like surface, but instead can have a rather irregular shape. Thus, measuring the LC curvature index may detect the topographical variation of anterior LC surface when evaluating the LC deformation. Second, the LC curvature index is independent from variations in the BMO location. Almost all of the previous studies used the BMO as a reference plane when measuring the LC depth. However, Johnstone et al.[24] recently reported that the BMO is located more posteriorly in older individuals, presumably due to age-related choroidal thinning. In addition, the BMO plane may fluctuate due to diurnal variations in choroidal thickness.[25–27] The LC curvature index, which is the difference between the mLCD and ALID, is independent of the location of BMO.

Increased IOP, or even IOP values within the normal range in patients with increased susceptibility to IOP-related stress, induces structural deformation (strain) of the load-bearing connective tissues and causes posterior bowing of the LC. This deformation induces numerous subsequent pathological changes in adjacent connective tissues, retinal ganglion cell axons, astrocytes, endothelium, and ONH blood flow, leading to a hyper-compliant and permanently remodeled LC structure. This series of processes may occur in the early stages of glaucoma.[28] Park et al.[29] recently compared LC depth according to glaucoma stage, and confirmed that LC posterior displacement occurred mostly in the early glaucoma stage and that there was no significant difference in LC depth between mild-to-moderate and severe glaucoma. The present data is consistent with the previous studies, in that LC curvature index values were increased in POAG eyes relative to healthy eyes but showed no difference between mild and moderate-to-advanced glaucoma. Considering all of the findings (previous and present) together, it can be concluded that LC posterior bowing as well as posterior displacement can occur in early stages of glaucoma.

**Fig 5:**
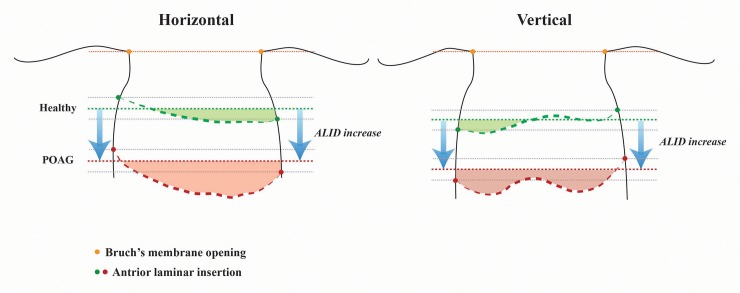
The schematic diagram showing the structural difference of lamina cribrosa (LC) between the eyes with primary open-angle glaucoma (POAG) and healthy individuals. Structural differences of the LC between POAG and healthy eyes are demonstrated. The orange dotted line corresponds to the reference plane of Bruch’s membrane openings. The thicker green and red dotted line indicates the anterior LC surface of healthy and POAG eyes, respectively. The thinner green and red dotted line presents the level of mean anterior laminar insertion depth (ALID). The green and red shaded area represents the relative posterior location of the anterior LC according to the mean ALID level of healthy and POAG eyes, respectively. Each shaded area reflects the degree of LC posterior bowing (LC curvature index). The ALID and LC posterior bowing (LC curvature index) are higher in POAG eyes than in healthy eyes. Note that vertical insertions are deeper than horizontal insertions. Additionally, the horizontal LC curvature index is larger than the vertical LC curvature index in both healthy and POAG eyes, due to the central hump-like structure in the vertical scan.

Our data showed that the horizontal LC curvature index was about two-fold larger than the vertical LC curvature index, whereas vertical ALID was greater than horizontal ALID. In other words, the LC inserts more posteriorly in the vertical than in the horizontal axis, though its architecture tells us that it is less posteriorly bowed in the vertical axis. This finding is consistent with Thakku et al.’s recent report characterizing anterior LC morphology in a healthy Indian population.[30] By measuring the curvature along 180 LC radial scan cross-sections, they demonstrated that the insertion points were deeper in the vertical axis than in the horizontal axis, whereas the curvature showed the W-shape in the vertical axis but the U-shape in the horizontal axis. Park et al.[31] identified the central horizontal ridge of the LC in healthy eyes using EDI SD-OCT. The location of this hump-like structure corresponded well with central retinal vessel trunk in the LC. Thus, the anterior LC contour features a “W-shape” in the vertical meridian whereas the horizontal scan shows a “U-shape” or “planar shape.” The central horizontal ridge of the LC may have contributed to the reduced LC curvature index values in the vertical scans compared to the horizontal scans in the present study (**Fig 5**).

Greater disc area was significantly associated with increased LC posterior bowing. Large disc size has been known to be associated with increased glaucoma susceptibility.[32] This might be due to increased IOP-related damage of the LC as per Laplace’s law for a given level of IOP. Large disc size also is associated with myopic eyes, which are susceptible to glaucomatous damage. Jonas and Budde showed higher glaucoma susceptibility in highly myopic eyes with large optic discs than in non-highly myopic eyes.[33] The present finding that myopic refraction showed marginal significance (*P* = 0.049) in terms of increased LC posterior bowing further supports the previous findings.

Greater vertical C/D ratio and mLCD along with smaller rim area were significantly associated with increased LC posterior bowing. These parameters are well known risk factors for glaucoma. In fact, the present data support recent findings on the association of increased LC curvature with larger vertical C/D ratio, increased LC depth, and smaller minimum rim width (BMO-MRW) in healthy Indian eyes.[30] Taken together, the findings suggest that optic disc cupping is highly associated with LC posterior bowing. This is further supported by a recent report of Wu et al., who evaluated longitudinal displacement of the ONH and anterior LC surfaces.[34] They found anterior as well as posterior displacement of the ONH and anterior LC surfaces and demonstrated a relatively strong association between ONH and anterior LC surface changes.

In the present study, average RNFL thickness tended to be thinner in eyes with increased LC posterior bowing. This structure/structure relationship between RNFL thickness and LC deformation has been raised previously. Jung et al.[35] reported increased LC depth associated with decreased average RNFL thickness. Ren et al.[36] demonstrated an age-dependent relationship between LC depth and RNFL thickness (but which did not attain statistical significance). Lee et al.[37] recently reported increased RNFL thickness progression associated with greater LC depth and thinner LC. The present findings further support the structure/structure relationship between LC posterior bowing and RNFL thickness.

Interestingly, neither untreated IOP nor IOP at examination was associated with the LC curvature index value. The IOP-related stress (force) present at a given level of IOP is determined by the three-dimensional (3D) ONH anatomy, and this generates IOP-related strain (tissue deformation), which depends on the material properties of the ONH.[1] Since these features of the ONH can vary significantly across individuals, the resulting changes in LC structure may also be diverse at a given level of IOP. This may result in the lack of a significant association between the IOP parameters and LC curvature index in the regression analysis. However, this should be interpreted with caution since the majority of glaucoma patients in the present study were POAG patients with untreated IOP in normal range. Further investigation of the relationship between IOP and LC curvature index should also include POAG patients that have high baseline IOP.

Even the POAG eyes had an IOP level lower than that of healthy eyes at the time of the OCT examination, they showed an increased LC curvature index in the present study. We speculate that this may be explained by the following hypotheses. First, once the connective tissues of the LC mechanically fail due to increased IOP, the remaining adjacent healthy LC suffers from increased IOP-related stress, even under a constant level of IOP. This may lead to progressive damage and remodeling of ONH even when IOP-lowering treatment has been applied. The increased LC curvature index in POAG eyes may reflect a permanent deformation of LC. Second, the LC in POAG eyes may be hyper-compliant, so that the degree of posterior deformation may be larger at a given level of IOP.[28, 38, 39]

The present study has some limitations. First, the LC curvature index measurement was performed only in the horizontal and vertical meridians. This might neglect the supero-temporal and infero-temporal regions of the LC, where glaucomatous RNFL defect most commonly occurs, on evaluation of its entire architecture. Thakku et al.[30] recently characterized anterior LC morphology by measuring curvature along 180 LC cross-sections. They found that the principal LC arc curvature is roughly perpendicular and aligned to the horizontal and vertical axes. Thus, cross-line scans might be sufficient for understanding generalized LC curvature. Further investigation using radial scans would allow for more precise assessment of LC posterior bowing. Second, the majority of the glaucoma patients (113 out of 123 patients) in the present study were POAG patients with untreated IOP ≤ 21 mmHg. Therefore, our conclusions should not be generalized to high-IOP glaucoma patients. The relationship between IOP and the LC curvature index needs to be evaluated with further recruitment of high-IOP glaucoma patients, which is ongoing in our study group.

In conclusion, the POAG eyes showed increased LC posterior bowing (LC curvature index) in both the horizontal and vertical meridians compared to healthy controls. Furthermore, the LC curvature index was significantly associated with structural ONH change but not with functional glaucoma severity (VF MD). Quantitative assessment of LC posterior bowing enabled by SS-OCT may therefore provide additional structural information when evaluating glaucomatous optic nerve head change.
